# Development of a *Chlamydomonas reinhardtii* metabolic network dynamic model to describe distinct phenotypes occurring at different CO_2_ levels

**DOI:** 10.7717/peerj.5528

**Published:** 2018-09-03

**Authors:** Daniela Alejandra Mora Salguero, Miguel Fernández-Niño, Luis Miguel Serrano-Bermúdez, David O. Páez Melo, Flavia V. Winck, Camila Caldana, Andrés Fernando González Barrios

**Affiliations:** 1 Grupo de Diseño de Productos y Procesos (GDPP), Department of Chemical Engineering, Universidad de Los Andes, Bogotá, Colombia; 2 Bioprocesses and Bioprospecting Group, Universidad Nacional de Colombia, Bogotá D.C., Colombia; 3 Laboratory of Regulatory Systems Biology, Department of Biochemistry, Institute of Chemistry, Universidade de São Paulo, São Paulo, Brazil; 4 Brazilian Bioethanol Science and Technology Laboratory, Brazilian Center for Research in Energy and Materials, Campinas, Brazil; 5 Max Planck Partner Group, Brazilian Bioethanol Science and Technology Laboratory, Brazilian Center for Research in Energy and Materials, Campinas, Brazil

**Keywords:** *Chlamydomonas reinhardtii*, CO_2_ fixation, Metabolomics, Biomass, Transcriptomics, Biotechnology, Metabolic network, Dynamic model

## Abstract

The increase in atmospheric CO_2_ due to anthropogenic activities is generating climate change, which has resulted in a subsequent rise in global temperatures with severe environmental impacts. Biological mitigation has been considered as an alternative for environmental remediation and reduction of greenhouse gases in the atmosphere. In fact, the use of easily adapted photosynthetic organisms able to fix CO_2_ with low-cost operation is revealing its high potential for industry. Among those organism, the algae *Chlamydomonas reinhardtii* have gain special attention as a model organism for studying CO_2_ fixation, biomass accumulation and bioenergy production upon exposure to several environmental conditions. In the present study, we studied the *Chlamydomonas* response to different CO_2_ levels by comparing metabolomics and transcriptomics data with the predicted results from our new-improved genomic-scale metabolic model. For this, we used *in silico* methods at steady dynamic state varying the levels of CO_2_. Our main goal was to improve our capacity for predicting metabolic routes involved in biomass accumulation. The improved genomic-scale metabolic model presented in this study was shown to be phenotypically accurate, predictive, and a significant improvement over previously reported models. Our model consists of 3726 reactions and 2436 metabolites, and lacks any thermodynamically infeasible cycles. It was shown to be highly sensitive to environmental changes under both steady-state and dynamic conditions. As additional constraints, our dynamic model involved kinetic parameters associated with substrate consumption at different growth conditions (i.e., low CO_2_-heterotrophic and high CO_2_-mixotrophic). Our results suggest that cells growing at high CO_2_ (i.e., photoautotrophic and mixotrophic conditions) have an increased capability for biomass production. In addition, we have observed that ATP production also seems to be an important limiting factor for growth under the conditions tested. Our experimental data (metabolomics and transcriptomics) and the results predicted by our model clearly suggest a differential behavior between low CO_2_-heterotrophic and high CO_2_-mixotrophic growth conditions. The data presented in the current study contributes to better dissect the biological response of *C. reinhardtii,* as a dynamic entity, to environmental and genetic changes. These findings are of great interest given the biotechnological potential of this microalga for CO_2_ fixation, biomass accumulation, and bioenergy production.

## Introduction

Atmospheric CO_2_ concentration has significantly increased over the last 100 years as a result of numerous anthropogenic activities. This has strongly contributed to climate change and global warming leading to a severe environmental crisis (Sayre, 2010). In the recent years, the use of photosynthetic organisms able to convert atmospheric CO_2_ into metabolites and other compounds of interest arose as an important alternative to mitigate such problem. In addition, CO_2_-fixing organism have been also shown to be a suitable platform for the production of inexpensive bulk chemicals with potential commercial interest using the atmospheric CO_2_ as a feedstock (Boyle & Morgan, 2009).

Among CO_2_-fixing organisms, aquatic photosynthetic microorganisms have gained increased attention since they perform almost 50% of global photosynthesis. Even when the diffusion of CO_2_ in aqueous solution has been reported to be a thousand times slower as compared to its diffusion on air (Moroney & Somanchi, 1999), several aquatic photosynthetic microorganisms have evolved CO_2_ concentrating mechanisms (CCMs) to adapt to their environment. These mechanisms increase photosynthetic productivity by scaling up the levels of inorganic carbon over the environmental levels of CO_2_ (Moroney & Ynalvez, 2007). One of these organisms is the algae *Chlamydomonas reinhardtii*, which is considered a promising organism with a high potential for CO_2_ fixation and bioenergy production. This well-known microorganism has been used as a model to study the cellular and biochemical response to a large number of environmental conditions including nutrient deprivation (Harris, 2001). For example, it has been shown that lipid accumulation is favored once *C. reinhardtii* is grown under conditions of nitrogen starvation (Scranton et al., 2015). Moreover, it has been shown that modifications in the culture conditions of *C. reinhardtii* significantly influence its final biomass (Rosenberg et al., 2008). Interestingly, this algae has been successfully photoautotrophically, heterotrophically, and mixotrophically cultivated (Rupprecht, 2009).

As explained above, *C. reinhardtii* have evolved CCMs in order to increase intracellular CO_2_ concentrations by using active C_i_-transport, thereby facilitating the rate of photosynthetic CO_2_ fixation even at low concentrations of external C_i_. Given that this microalga must overcome the 1000-fold slower diffusion of CO_2_ in water than in air, the active transport and accumulation of C_i_, either as CO_2_ or as HCO_3_^−^, plays a critical role (Moroney & Ynalvez, 2007). Carbonic anhydrases (CAs) has been shown to play a major role in the CCM because they catalyze the interconversion of CO_2_ and HCO_3_^−^, thereby maintaining and accelerating transport across the plasma membrane and to chloroplasts (Fang et al., 2012).

Significant advances have been made in order to elucidate the *Chlamydomonas’* CCM, including not only components of C_i_ uptake systems, but additional regulatory factors (Yamano & Fukuzawa, 2009). For example, the expression of these C_i_ transporters was shown to be regulated by several transcription factors that are responsive to changes in the environmental CO_2_ concentration (Winck, Páez Melo & González Barrios, 2013). Under CO_2_ limiting conditions, cells capture more C_i_ by altering the expression of thousands of genes, which, in turn, may be involved in the acceleration and enhancement of C_i_ acquisition. In other words, low CO_2_ concentrations has been reported to induce the *C. reinhardtii* CCM, which have facilitated the identification of several additional CCM-related genes by determining their expression level under limiting CO_2_ (lower than 0.05%) as compared to high CO_2_ (1% to 5%) (Fang et al., 2012). Moreover, additional factors such as the cooperation between light and carbon signaling have been shown to be also necessary for the modulation of CCM-related genes (Winck, Páez Melo & González Barrios, 2013). In this regard, our group has previously identified a vast range of genes and proteins that integrate carbon-related mechanisms (Winck, Páez Melo & González Barrios, 2013).

*C. reinhardtii* shows three CO_2_ acclimation states: high concentration (>5% [CO_2_]), low concentration (0.04%–0.03% [CO_2_]), and very low concentration (<0.01% [CO_2_]) (Fang et al., 2012). Therefore, an understanding of the metabolic pathways involved in the acquisition and accumulation of CO_2_ over these stages is fundamental for the identification of metabolites that contribute to acclimation, cellular growth, and biomass production in response to changes in environmental CO_2_ concentrations (Boyle & Morgan, 2009). In this regard, *in silico* analysis to model these metabolic pathways and its behaviors under variable conditions became a relevant strategy. Experimental validation of these models improves their predictive capabilities because not all metabolic pathways are necessarily activated in response to a specific cellular response or adaptation (Schmidt et al., 2010). Therefore, experimental data are essential for describing the substrates and products present in the cell. In this regard, the information obtained through experimental approaches from OMICS (i.e., transcriptomics, proteomics, and metabolomics) can be integrated into a reconstructed metabolic network model by the inclusion of new constraints as described by Kauffman, Prakash & Edwards (2003). Application of these restrictions reduces the solution space of possible phenotypes, thereby allowing a more accurate reconstruction and characterization of the metabolic network (Blazier & Papin, 2012). All these strategies can improve our capacity to predict the cellular behavior and identify pathways involved in biomass accumulation based on the observation of effects in the outputs. This is crucial to develop new strategies for improving the ability of *C. reinhardtii* to acquire and accumulate carbon (Winck, Páez Melo & González Barrios, 2013).

This work arose from our previously developed network for *C. reinhardtii* (Winck et al., 2016) and compares metabolomics and transcriptomics data with results predicted by our reconstructed genome-scale model (GSM) at steady-state and dynamic conditions at different CO_2_ levels. Our main goal was to improve the capacity to predict metabolic routes for biomass accumulation in *C.  reinhardtii*. For this purpose, we first curated the reconstructed model for subsequent analysis mainly of pathways sensitive to CO_2_ and genes related to carbon uptake and accumulation applying a steady-state and dynamic model. Then, we generated a phenotypically accurate and predictive computational model for *C. reinhardtii*, which represents a major advance over previous models (Imam et al., 2015; Winck et al., 2016).

Our predicted results improved our understanding of the biological response of *C. reinhardtii,* as a dynamic entity, to environmental changes. So far, almost all physical networks reported in the literature have been examined under a single static condition. Nevertheless, biological systems are highly dynamic and static approaches have failed to identify and integrate parameters that are condition specific (Ideker & Krogan, 2012). Considering the effects of CO_2_ concentration from a metabolic dynamic perspective is of great significance given the well-known biotechnological potential of *C. reinhardtii* for CO_2_ fixation, biomass accumulation, and bioenergy production.

## Materials and Methods

### Genome-scale metabolic network reconstruction

GSM reconstruction involved the following steps: (i) Identification of metabolic pathways and membrane transport reactions based on the genomic annotation, enzyme homology, and experimental observations. This step used databases such as KEGG, ModelSEED, TCD, and TransportDB, among others; (ii) Development of equations for biomass components based on physiological data or comparison between similar species; (iii) Identification of incomplete pathways and transport reactions of missing metabolites through semi-automated methods, such as reverse engineering, or automated methods, such as *GapFind* and *GapFilling* (Kumar, Dasika & Maranas, 2007; Senger & Papoutsakis, 2008; Serrano-Bermúdez, 2016). In this work, we first improved our previously developed network for *C. reinhardtii* (Winck et al., 2016), which required the extraction of all the available metabolic information (e.g., reactions, compounds, pathways, genes, enzymes) from the following public databases: Plant Metabolic Network (ChlamyCyc 5.0, 2015), KEGG (Kanehisa Laboratories, 2016), BioCyc (BioCyc Database Collection, 2016), and ModelSEED (ModelSEED, 2015). In addition, the *i*Cre1355 model (Imam et al., 2015) was used to enhance our reconstruction. Manual curation was required through the comparison between models in order to verify the presence of novel substrates, products, and reactions by comparing several models. Manual gap-filling was carried out to fill additional gaps using literature and experimental data from different databases. Thus, manual curation was used to resolve issues related to reaction stoichiometries of reactions not found in the databases, or genes coming from extensive bioinformatic analysis not yet submitted. Reactions directionality were determined as reported by Mavrovouniotis and co-workers (Mavrovouniotis, 1990). Each metabolite in our GSM model may have one or more designations denoting its cellular compartment, which allowed the inclusion of exchange reactions designed to connect novel metabolites in their cellular compartments.

### *Chlamydomonas reinhardtii* kinetic modeling

Kinetic parameters associated with substrate consumption at different growth conditions (i.e., 0% CO_2_-heterotrophic and high CO_2_-mixotrophic) were used as additional constraints in the dynamic model. Then, based on the growth conditions, we constructed two *Chlamydomonas* kinetic models: (i) heterotrophic growth and (ii) mixotrophic growth.

#### Heterotrophic dynamic model for cell growth and substrate consumption

In this work, we used the Haldane model, which includes terms to describe the effects of a nutrient on the growth rate at high concentrations (Zhang, Chen & Johns, 1999; Lee, Jalalizadeh & Zhang, 2015).

\begin{eqnarray*}& \mu ={\mu }_{max} \frac{[Ac]}{{K}_{s}+[Ac]+ \frac{{ \left[ Ac \right] }^{2}}{{K}_{i}} } \end{eqnarray*}

\begin{eqnarray*}& \frac{dX}{dt} ={\mu }_{max} \frac{[Ac]}{{K}_{s}+[Ac]+ \frac{{ \left[ Ac \right] }^{2}}{{K}_{i}} } X \end{eqnarray*}

\begin{eqnarray*}& \quad - \frac{d \left[ Ac \right] }{dt} = \frac{1}{{Y}_{x}} \frac{dX}{dt} +mX \end{eqnarray*}

where *X* is algal density or concentration [*g*∕*L*], [*Ac*] is substrate (acetate) concentration [*g*∕*L*], *t* is time [*h*], *μ*_*m*_ is maximum specific growth rate [1∕*h*], *K*_*s*_ is the acetate saturation constant [*g*∕*L*], *K*_*i*_ acetate inhibition constant [*g*∕*L*], *Y*_*x*_ is yield constant on acetate $ \left( \frac{g Alage produced}{g of nutrient} \right) [g/g]$, and *m* is the maintenance energy coefficient.

#### Mixotrophic dynamic model for cell growth and substrate consumption

The behavior of microalgal cultures was described with a set of nonlinear algebraic-differential equations deduced from mass balance considerations for both liquid and gaseous phases, assuming well-mixed conditions, and following the Haldane model (Tebbani et al., 2013).

\begin{eqnarray*}& & \frac{dX}{dt} ={\mu }_{max} \frac{ \left[ {C}_{DIC} \right] }{{K}_{s{C}_{DIC}}+[{C}_{DIC}]+ \frac{{ \left[ {C}_{DIC} \right] }^{2}}{{K}_{i{C}_{DIC}}} } \mathrm{ \ast } \frac{ \left[ Ac \right] }{{K}_{sAC}+ \left[ Ac \right] + \frac{{ \left[ Ac \right] }^{2}}{{K}_{iAC}} } X \end{eqnarray*}

\begin{eqnarray*}& & \frac{d \left[ {C}_{DIC} \right] }{dt} =- \frac{1}{{M}_{x}} \frac{dX}{dt} + {N}_{C{O}_{2}} \end{eqnarray*}

\begin{eqnarray*}& & \frac{d \left[ Ac \right] }{dt} =- \frac{1}{{Y}_{x}} \frac{dX}{dt} + mX \end{eqnarray*}

\begin{eqnarray*}& & {N}_{C{O}_{2}}= {k}_{L}{a}_{C{O}_{2}} \left( \frac{P}{{H}_{C{O}_{2}}} {y}_{out}^{C{O}_{2}}-[C{O}_{2}] \right) \end{eqnarray*}

\begin{eqnarray*}& & \left[ C{O}_{2} \right] = \frac{ \left[ {C}_{DIC} \right] }{(1+{K}_{1}1{0}^{pH}+{K}_{1}{K}_{2}1{0}^{2pH})} \end{eqnarray*}

where [*C*_*DIC*_] is dissolved inorganic carbon concentration [*mol*∕*L*], *K*_*sC*_*DIC*__ is the *C*_*DIC*_ saturation constant [*mol*∕*L*], *K*_*iC*_*DIC*__ is the *C*_*DIC*_ inhibition constant [*mol*∕*L*], *M*_*x*_ is the C-mole mass, *N*_*CO*_2__ is the volumetric mass transfer rate for *CO*_2_ $ \left[ \frac{mol}{{m}^{3}\ast h} \right] $, *k*_*L*_*a*_*CO*_2__ is the volumetric mass transfer coefficient [1∕*h*], *P* is the pressure $ \left[ Pa \right] $, *H*_*CO*_2__ is the *CO*_2_Henry constant [*Pa*∗*m*^3^∕*mol*], ${y}_{out}^{C{O}_{2}}$ is the *CO*_2_ molar fraction in the output gas, and *K*_1_ and *K*_2_ are equilibrium constants.

### Constraint-based modeling

#### Flux Balance Analysis (FBA)

FBA assumes that metabolic networks achieve a steady state that is constrained by the stoichiometry and that they keep on self-adjusting and optimizing in a changing external environment. This method then uses a mathematical model to simulate the network self-adjustment (Kauffman, Prakash & Edwards, 2003; Chen, Wang & Wei, 2010). Here, we reconstructed our model following the same parameters described in our previous work (Winck et al., 2016), but for all new tested conditions (i.e., photoautotrophic, heterotrophic, and mixotrophic conditions).

Then, CO_2_ fluxes were changed in order to determine the impact of the different tested CO_2_ concentrations supply at the steady state of cell metabolism. The biomass function was exactly the same as described in our previous study (Winck et al., 2016). The LBs and UBs of the reactions were fixed according to standard values, the criteria of the free Gibb’s energy at 27 °C and pH 7, and the database information. A maximization of the biomass reaction flux was performed, and the optimization problem was resolved to employ the GAMS^®^ (General Algebraic Modeling System: https://www.gams.com/) distribution 23.9.5 using FBA by solving the linear programming problem with constraints set as follows:

\begin{eqnarray*}& & ma{x}_{z}{c}^{T}v(1) \end{eqnarray*}

\begin{eqnarray*}& & \text{subject to} \mathbi{S}v=\mathbf{0} \end{eqnarray*}

\begin{eqnarray*}& & LB\leq v\leq UB \end{eqnarray*}

${c}^{T}\in {R}^{n}{|}{c}^{T}=[000\ldots 1\ldots 000]pos \left( 1 \right) =Biomass reaction$

\begin{eqnarray*}& & v\in {R}^{n} \end{eqnarray*}

\begin{eqnarray*}& & \mathbi{S}\in {R}^{mxn} \end{eqnarray*}

*LB* ∈ *R*^*n*^, *UB* ∈ *R*^*n*^

where ***S*** is the stoichiometric matrix, ***v*** is the flux vector, *LB* is the lower bound, *UB* is the upper bound, *c* is a vector of zeros that sets the objective function, *m* is the number of metabolites, *n* is the number of reactions, and $pos \left( 1 \right) $ is the objective function (biomass) (Gianchandani, Chavali & Papin, 2010). Finally, those reactions that changed at different CO_2_ levels were identified by FBA. In that way, we were able to determine candidate metabolic pathways of relevance for a CO_2_ response.

#### Flux Variability Analysis (FVA)

Linear program (LP) problems arising in FBA can have multiple solutions with the exact same optimal value for the objective function and still satisfy all the constraints. The number of these solutions determines the dimensionality of the alternative optimal space. These variables can be identified using flux variability analysis (FVA), where the flux of each reaction in the network is maximized and minimized, one at a time, while the optimal value is obtained from FBA (Mahadevan & Schilling, 2003; Maranas & Zomorrodi, 2016). Here, the optimization problem was as follows:

\begin{eqnarray*}& & \text{maximize(and minimize)}\;z={v}_{j} \end{eqnarray*}

\begin{eqnarray*}& & \text{subject to} \end{eqnarray*}

\begin{eqnarray*}& & \sum _{j\epsilon J}{S}_{ij}{v}_{j}=0,\forall i\epsilon I \end{eqnarray*}

\begin{eqnarray*}& & L{B}_{j}\leq {v}_{j}\leq U{B}_{j},\forall j\epsilon J \end{eqnarray*}

\begin{eqnarray*}& & {v}_{biomass}=f{v}_{biomass}^{max} \end{eqnarray*}

\begin{eqnarray*}& & {v}_{j}\epsilon \mathbb{R},\forall j\epsilon J. \end{eqnarray*}

FVA was then used to identify blocked reactions in a metabolic network under a given environmental and growth condition. Blocked reactions were reactions with both a minimum and maximum flux value equal to zero under the examined condition. The relevance of identifying blocked reactions was: (i) that they could be used as a basis for improving the quality of metabolic network reconstruction by helping to identify gaps, or (ii) that they could significantly reduce the search space of FBA by pre-setting their value to zero (Maranas & Zomorrodi, 2016).

#### Thermodynamically infeasible cycles (TICs)

The loop law states that around a metabolic loop all the thermodynamic driving forces must add up to zero. In other words, a net flux cannot exist around a closed cycle in a network at steady state (Schellenberger, Lewis & Palsson, 2011). By using the FVA method a loopless analysis was performed, which consisted of a sequential maximization and minimization of each reaction in the model. Those reactions where the range (max *v*_*i*_ -min *v*_*j*_) differed by >10^−6^ were defined as loop reactions (Schellenberger, Lewis & Palsson, 2011). The loopless condition used to identify all cycles can be written as the condition where *G* is a vector of energies for each reaction. All loops can be expressed as a linear combination of the null basis of ***S*** matrix in the form *v* = *Nxα*, where *N* = *null*(*S*), and *αare* weights. As described above, the loopless condition generates an LP problem. The driving force of each reaction was then indicated by the vector of continuous variables (*G*_*i*_) .This quantity can be thought of as being analogous to the Δ*G* of each reaction. As a loop is entirely defined by the direction of the flux distribution (Schellenberger, Lewis & Palsson, 2011), if *NxG*_*i*_ = 0, no loop must be present. This methodology consisted of the detection of reactions with maximum and/or minimum possible values on fluxes (1,000 and −1,000 (mmol/g*h)) using the FVA. Subsequently, these reactions and the metabolites involved are expressed in matrix form, and the null space of the matrix is calculated. Each thermodynamically infeasible cycle (TIC) is thus detected with the reactions involved. The TICs are solved through manual revision of reversibility or irreversibility of the reactions involved, removal of repeated reactions, or as a last option, the blocking of some reactions. Finally, this procedure is repeated until the null space is zero; thus, the GSM reconstructed model is cured (Maranas & Zomorrodi, 2016). Here, the optimization problem (FVA) was resolved using GAMS^^®^^ (General Algebraic Modeling System) distribution 23.9.5. Computation for obtaining the null space of S was performed in MATLAB^®^ version 2015b (MathWorks, Natick, MA, USA) followed by manual correction of reversibility in the involved reactions according to the databases sources.

#### *Chlamydomonas reinhardtii* metabolic network dynamic simulation

A variant of FBA, called dynamic flux balance analysis (DFBA), allows a description of the transience of metabolism. DFBA can be implemented in two ways: by a dynamic optimization approach (DOA), which requires solving a nonlinear program (NLP), and by a static optimization approach (SOA), which requires solving an LP (Gianchandani, Chavali & Papin, 2010). On one hand, DOA involves optimization over the entire time period of interest to obtain time profiles of fluxes and metabolite levels (Mahadevan, Edwards & Doyle, 2002). On the other hand, SOA distributes the time into different time intervals and solve the immediate optimization problem at the beginning of each of these intervals. This allows to predict the fluxes of each time point, which is followed by integration over the interval to compute species concentrations over time (Mahadevan, Edwards & Doyle, 2002; Gianchandani, Chavali & Papin, 2010). Here, we performed the dynamic model using SOA that involved kinetic parameters as additional constraints, and the optimization problem was resolved using GAMS^^®^^ (General Algebraic Modeling System) distribution 23.9.5 by setting the following:

\begin{eqnarray*}& & max\sum _{j=1}^{N}{c}_{j}\cdot {v}_{j}(t) \end{eqnarray*}

\begin{eqnarray*}& & \text{subject to} \end{eqnarray*}

\begin{eqnarray*}& & {z}_{i} \left( t+\Delta t \right) ={z}_{i} \left( t \right) -\sum _{j=1}^{N}{S}_{ij}\cdot {v}_{j} \left( t \right) \cdot X \left( t \right) \cdot \Delta t \forall i\epsilon 1,\ldots ,{M}_{extracellular} \end{eqnarray*}

\begin{eqnarray*}& & X \left( t+\Delta t \right) =X \left( t \right) +\mu \cdot X \left( t \right) \cdot \Delta t \end{eqnarray*}

\begin{eqnarray*}& & \sum _{j=1}^{N}{S}_{ij}\cdot {v}_{j}=0 \forall i\epsilon 1,\ldots ,{M}_{extracellular} \end{eqnarray*}

\begin{eqnarray*}& & {v}_{j}^{min}\lt {v}_{j} \left( t \right) \lt {v}_{j}^{max} \forall j\epsilon 1,\ldots ,N \end{eqnarray*}

\begin{eqnarray*}& & \hat {c} \left( {z}_{i} \left( t \right) ,{v}_{j} \left( t \right) \right) \leq 0 \end{eqnarray*}

\begin{eqnarray*}& & {z}_{i} \left( t \right) \geq 0\quad {z}_{i} \left( {t}_{0} \right) ={z}_{i,0} \forall i\epsilon 1,\ldots ,{M}_{extracellular} \end{eqnarray*}

\begin{eqnarray*}& & X \left( t \right) \geq 0 X \left( {t}_{0} \right) ={X}_{0} \end{eqnarray*}

\begin{eqnarray*}& & \Delta t= \frac{{t}_{f}-{t}_{0}}{G}  \forall G\epsilon 0\ldots G \end{eqnarray*}

\begin{eqnarray*}& & \forall {t}_{g}\epsilon [{t}_{0},{t}_{f}] \end{eqnarray*}

where *z*_*i*_ is the extracellular metabolite concentration (based on the components of the culture media), *z*_*i*,0_ and *X*_0_ are initial conditions for extracellular metabolite and algal concentrations, respectively, *X* is the algal concentration, *μ* is the specific growth rate, *c*_*j*_ is the reaction weight, $\hat {c} \left( z,v \right) $ is a vector of nonlinear constraints (i.e., kinetics parameters associated with substrate consumption), *t*_0_ and *t*_*f*_ are the initial and the final times, respectively, and *G* is the number of intervals in which the time is discretized.

### Cell strain and culture conditions

The *Chlamydomonas reinhardtii* strain CC503 cw92mt+ (Chlamydomonas Resource Center University of Minnesota, USA) was cultivated in tris-acetate-phosphate (TAP) medium (Anderson, 2005): phosphate solution (1 ml) (K_2_HPO_4_, 28.8 g 100 ml^−1^; KH_2_PO_4_, 14.4 g 100 ml^−1^), salts (25 ml) (NH_4_Cl, 15g L^−1^; MgSO_4_ 7H_2_O, 4g L^−1^; CaCl_2_ 2H_2_O, 2g L^−1^), trace elements (1 ml) (H_3_BO_3_,1.14 g 100 ml^−1^; ZnSO_4_ 7H_2_O, 2.2 g 100 ml^−1^; MnCl_2_ 4H_2_O, 0.5 g 100 ml^−1^; FeSO_4_ 7H_2_O, 0.5 g 100 ml^−1^; CoCl_2_ 6H_2_O, 0.16 g 100 ml^−1^; CuSO_4_ 5H_2_O, 0.16 g 100 ml^−1^; (NH_4_)_6_Mo_7_O_24_ 4H_2_O, 0.11 g 100 ml^−1^; EDTA 5 g 100 ml^−1^), acetic acid (1 ml), and tris (2.42 g/L) at 27 °C in heterotrophic and mixotrophic growth conditions, under different CO_2_ concentrations [0% and 10% CO_2_, respectively], in a R’ALF Plus bioreactor (Bioengineering, Inc., Cambridge, MA, USA) under continuous illumination. Cell growth, bioreactor experiments and counting of cells were performed as previously described in Winck et al. (2016).

### Total biomass measurement

Cells were grown in both heterotrophic (0% CO_2_ and acetic acid as a carbon source) and mixotrophic (10% CO_2_ and acetic acid as carbon sources) growth conditions. Then, the total biomass was determined based on dry weights following the method described in our previous work (Winck et al., 2016).

### Gene expression analysis by quantitative RT-PCR

Genes were selected based on our previously reported sensitivity analysis and included genes related to CCM, the Calvin Cycle, glycolysis/gluconeogenesis and glyoxylate/dicarboxylate metabolism (Winck et al., 2016). We performed a new sensitivity analysis using FBA and DFBA to contrast with the previous results and to describe other pathways involved using expression data (i.e., transcriptomic data). Cell pellets from 45 mL cell culture aliquots were collected on the last cultivation day in both growth conditions (heterotrophic and mixotrophic). Then, the total RNA was extracted by using the RNeasy Plant Mini Kit (QIAGEN 2012) and subsequently treated with TURBO DNAase (Ambion and Life Technologies, 2012) to remove remaining DNA. Synthesis of cDNA was performed using the iScript cDNA Synthesis Kit (BioRad, Hercules, CA, USA) according to the manufacturer’s instructions. The actin gene [ACT] was used as a reference gene. All the cDNA samples were amplified in 96-well plates in Agilent Technologies Stratagene Mx3005P system. Real-time PCR was performed used the same conditions as described in Winck et al. (2016). For each gene, a relative expression ratio was determined as previously described by Schmittgen and Livak (Schmittgen & Livak, 2008). PCR efficiencies were determined as previously described (Winck et al., 2016).

### Metabolomics analysis

Cell pellets from a culture in stationary phase were collected from the 0% CO_2_-heterotrophic and high CO_2_-mixotrophic cultures and metabolites were extracted and treated as previously described in Winck et al. (2016). Metabolites derivatization, GC-TOF-MS analysis, chromatogram analysis, peak detection was also performed used the methods previously described (Winck et al., 2016).

## Results

### Significantly improved and robust genome-scale metabolic network model of *C. reinhardtii*

Based on our previously reconstructed network and biomass reactions (Winck et al., 2016), we initially performed a curation process by reverse engineering. This allows us to identify some inconsistencies, such as disruptions in the balance of proton (H^+^) charges, disruptions in the balance of water consumption and production in some reactions, the oxygen requirement for growth, and the presence of the same reaction twice but one of them was the reverse reaction, among other factors. Initially, our model consisted of 3554 reactions and 2,342 metabolites, in contrast to *i*Cre1355, which has 2,394 reactions and 1,845 metabolites (Imam et al., 2015). A cross-referencing process with *i*Cre1355 allowed us to identify 172 new reactions, including reactions related to exchange, transport, amino acid synthesis, oxidative phosphorylation, and fatty acid biosynthesis, among others. Manual examination of KEGG and ModelSEED databases was used to link some products by applying gap filling. The reactions were compartmentalized into ten compartments: extracellular, cytosol, chloroplast, mitochondria, nucleus, eyespot, flagellum, glyoxysome, thylakoid lumen, and Golgi apparatus. Finally, we generated a new comprehensive genome-scale network reconstruction for *C. reinhardtii*, which consisted of 3,726 reactions and 2,436 metabolites (Data S1, S2 and S3).

**Figure 1:**
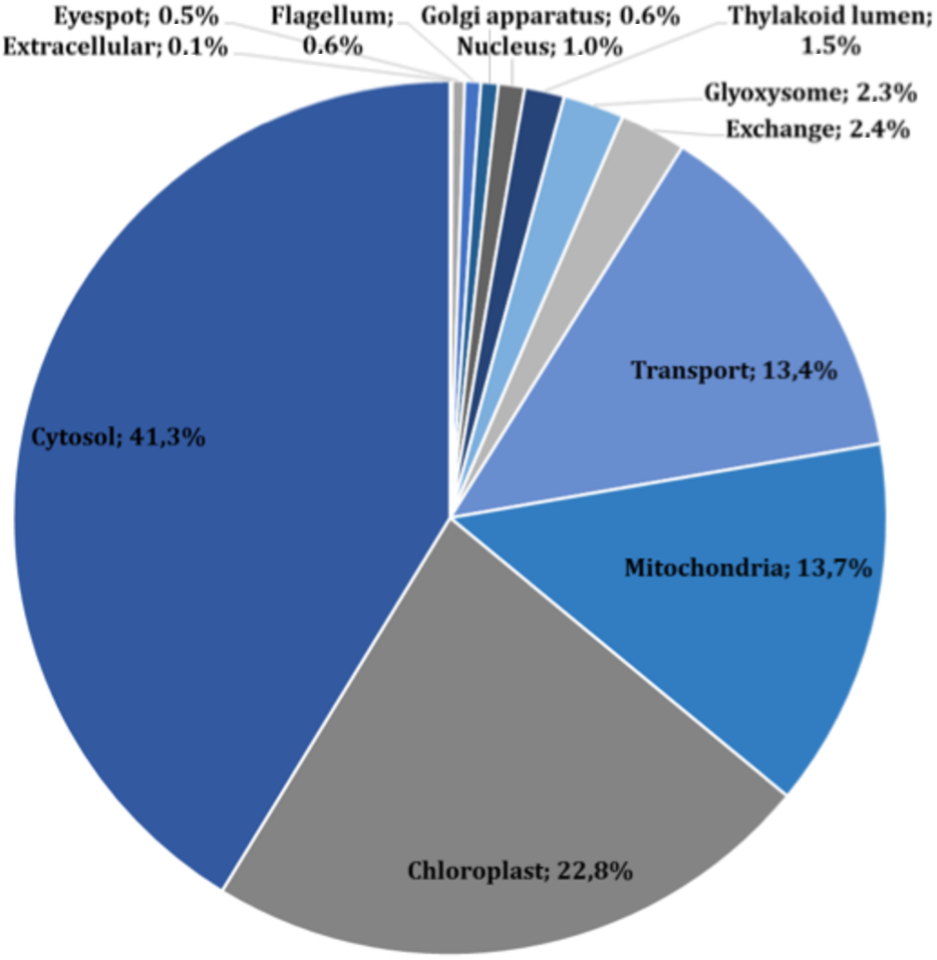
Subcellular localization of all reactions in the current reconstructed model (including exchange and transport reactions).

**Figure 2:**
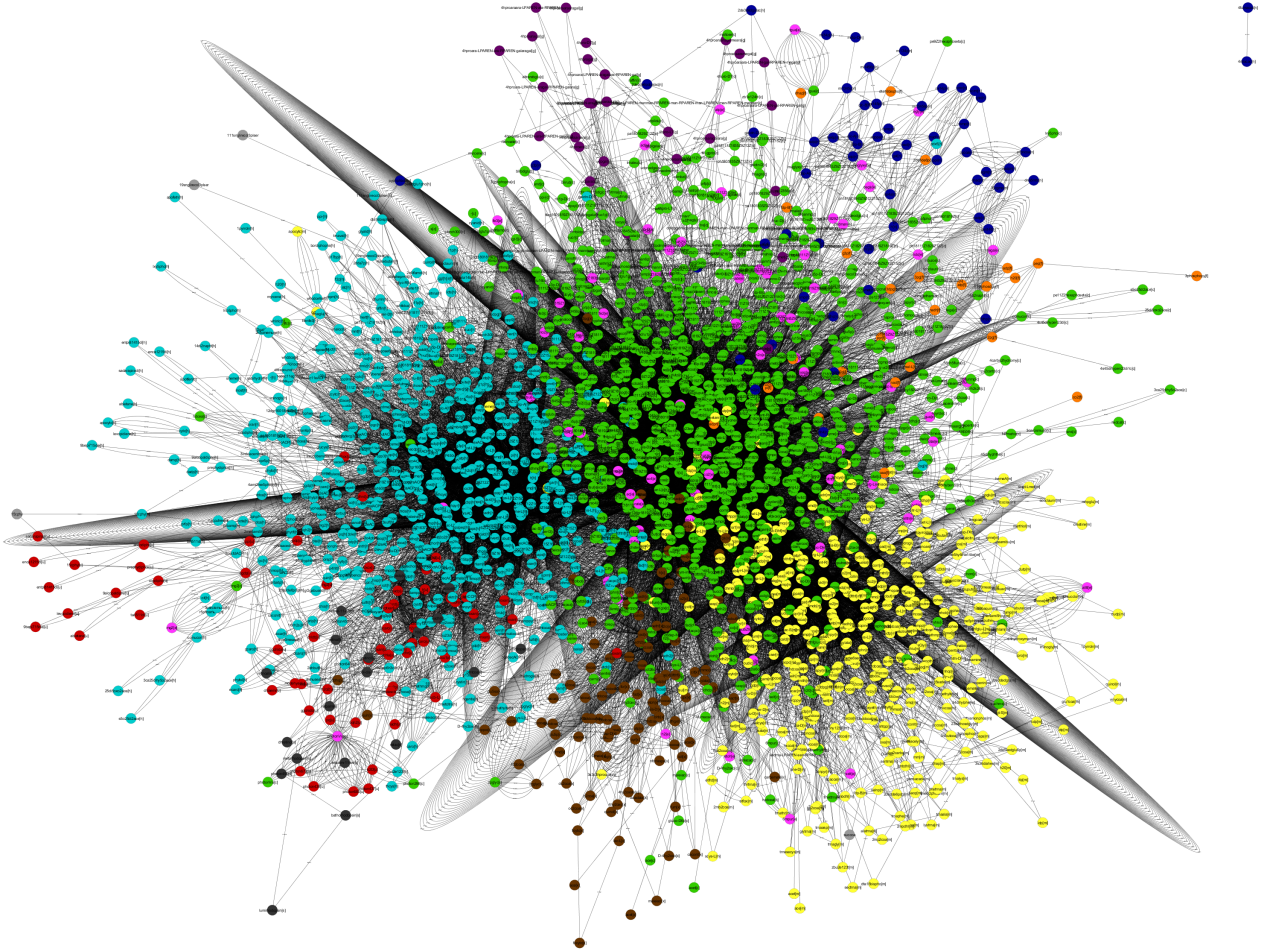
Map *Chlamydomonas reinhardtii.* Metabolic Network model. (Nodes: Metabolites, Edges: Reactions). Green, cytosol; light blue, chloroplast; yellow, Mitochondria; maroon, glyoxysome; orange, flagellum; red, thylakoid lumen; pink, Extracellular; dark blue, Nucleus; violet, golgi apparatus; gray, eyespot. This graphic map was performed in the visualization software Cytoscape (http://www.cytoscape.org/).

The main characteristics of our GSM model are summarized in Table 1. This model exhibits increases of 5% in the number of reactions over our previous model, and 56% in the number of reactions over the *i*Cre1355 model, as well as increases of 4% and 32% in the number of metabolites over the previous model and the *i*Cre1355 model, respectively. The new model we are presenting here also has 1,416 unique metabolites, which allows us to encompass a broader range of metabolic functions. As shown in Fig. 1, all reactions presented in the new reconstructed model were distributed across several compartments, including transport and exchange reactions. Compartmentalization was necessary to guarantee the location and viability of reactions as reported in databases and literature (Petersen et al., 2011; Winck et al., 2016). The interconnections in the current metabolic network are shown in Fig. 2. As in the previously developed model and the *i*Cre1355 model, the reactions in the current model are distributed over nine intracellular compartments and the extracellular space, with the majority of reactions localized to the cytosol, chloroplast, and mitochondria. Interestingly, the chloroplast was shown to be highly relevant in light-driven metabolism due to its relative centrality (Figs. 1 and 2). In fact, the chloroplast represents 25% of the total reactions in the network. These compartments involve crucial pathways for photoautotrophic growth, including photosynthesis, chlorophyll synthesis, and the mechanism for phototaxis (Chang et al., 2011).

**Table 1:** Comparison of components in the models.

**Components**	**Previous model**	**iCre1355**	**Current model**
**Reactions**	**3,554**	**2,394**	**3,726**
Transport	479	426	498
Exchange	72	55	91
Compartments	10	10	10
**Metabolites**	**2,342**	**1,845**	**2,436**
Unique metabolites	1,382	1,133	1,416
Cytosol	955	730	1,008
Chloroplast	626	478	638
Mitochondria	388	308	423

**Figure 3:**
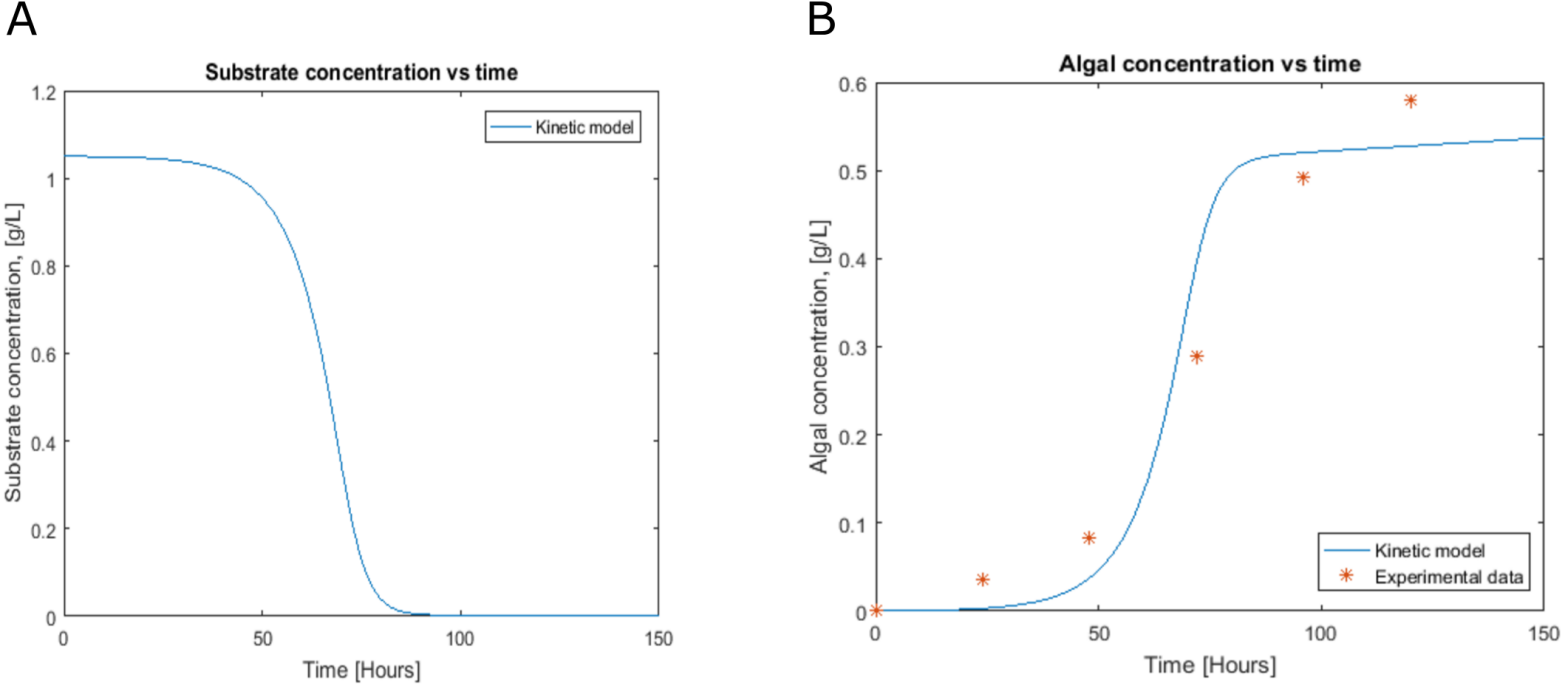
Profiles predicted by kinetic modeling at heterotrophic growth conditions (A) acetate consumption and (B) cell concentration.

**Figure 4:**
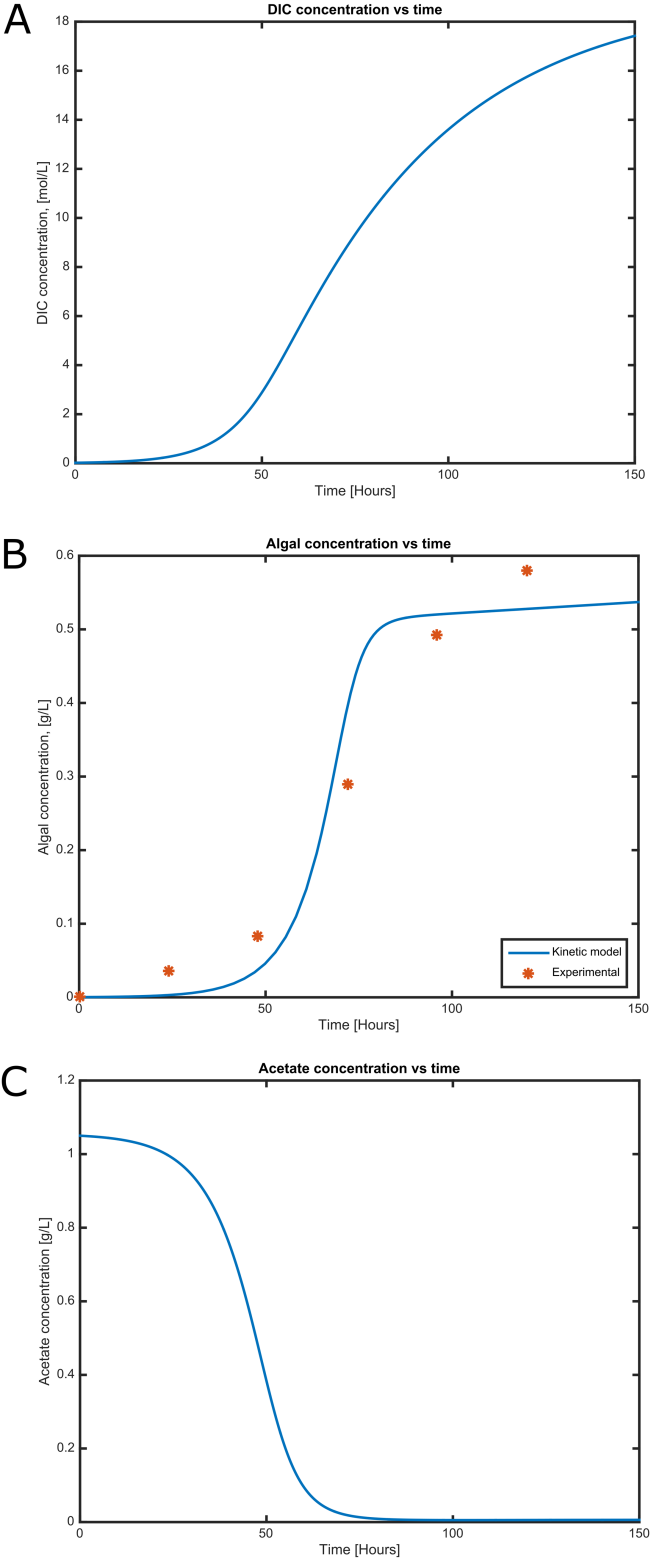
Time courses of (A) dissolved inorganic carbon concentration, (B) cell concentration and (C) acetate consumption in mixotrophic growth conditions of *C. reinhartii* in batch culture.

### Cell growth and substrate consumption behavior

Cell growth and substrate consumption profiles were obtained in batch culture under heterotrophic and mixotrophic conditions in order to utilize them as restrictions for the dynamic model (Figs. 3 and 4). pH variations ranging from 6.3 and 7.5 were chosen, based on previous kinetic modeling for the heterotrophic growth of *C. reinhardtii* in batch cultures using acetate as carbon source and ammonium as nitrogen source (Zhang, Chen & Johns, 1999). A lag phase of around 10 hours was observed and acetate was completely exhausted after 70 hours. The resulting cell concentration at heterotrophic conditions was around 0.55 g/L for the model and 0.58 g/L in our experimental data, which was in the same range and similar to the values reported by others (0.44 g/L) (Zhang, Chen & Johns, 1999). Likewise, the response of dissolved inorganic carbon concentration agreed with previous measurements of CO_2_ fluxes in *Chlamydomonas* under similar conditions (Badger, Palmqvist & Yu, 1994). The increasing cell concentration under mixotrophic conditions was due to the presence of both carbon sources (acetate and CO_2_) and the continuous input of CO_2_ into the media.

### The curated genome-scale metabolic network model of *C. reinhardtii* is highly sensitive under both steady-state and dynamic conditions

The guarantee of a robust and sensitive model to predict metabolic fluxes and to use in subsequent dynamic simulations required the correction of all TICs, along with a balancing of H^+^ charge and water consumption in the entire network. A total of 187 loops were identified and many cycles were corrected and eliminated to finally generate a metabolic network curated without any thermodynamically infeasible cycle.

**Figure 5:**
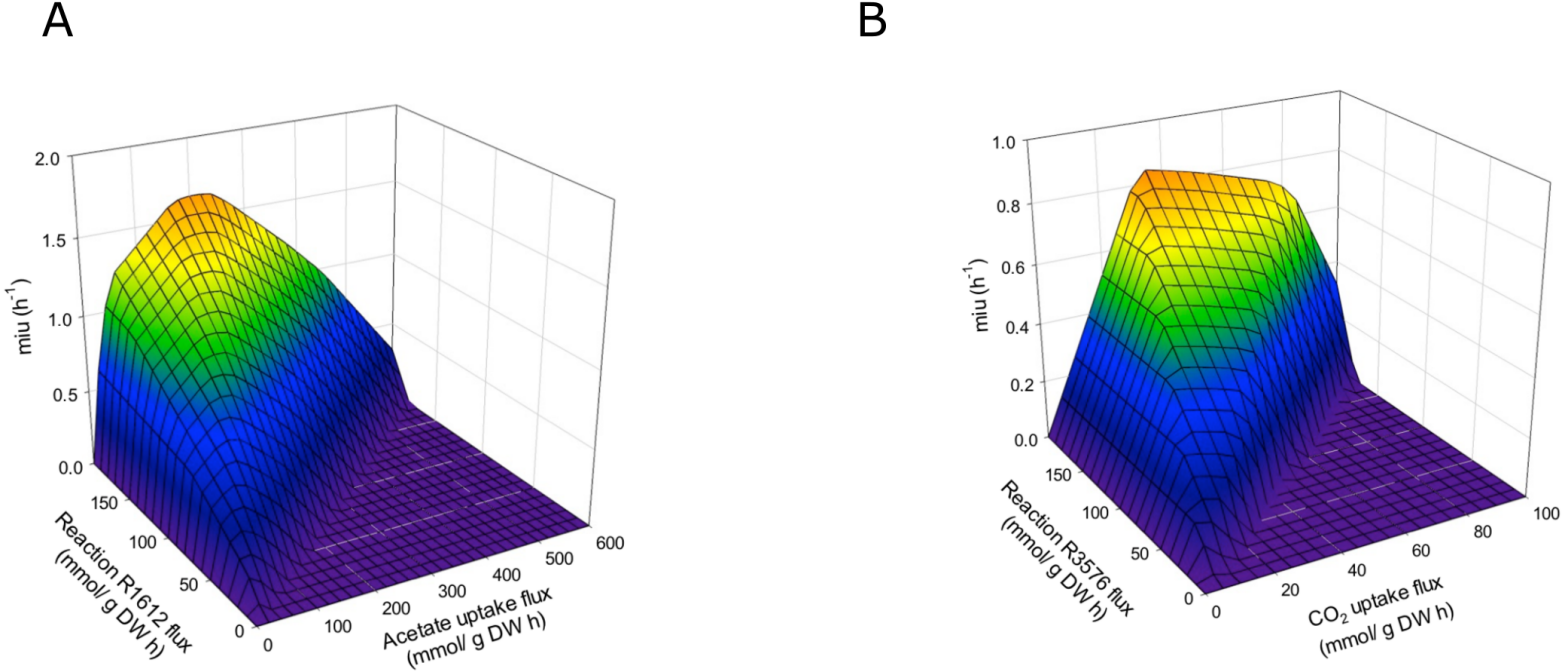
Phenotypic phase planes. Response surfaces of growth rates predicted by FBA in function to (A) Heterotrophic growth: Flux acetate and flux of reaction R1612 (ATP production in mitochondria). (B) Photoautotrophic growth: Flux CO2 and flux reaction R3576 (ATP production in chloroplast).

The final reconstructed model was used along with the FBA to predict the flux distribution in *C. reinhardtii* under photoautotrophic (using light and CO_2_ as energy and carbon sources), heterotrophic (using acetate as carbon source), and mixotrophic (using both acetate and CO_2_ as carbon sources, and light) growth conditions. This new sensitivity analysis implemented FBA and DFBA to describe new pathways using transcriptomic data, which provided a reference for optimizing biomass production upon different growth conditions. The predictions were performed with maximization of biomass. The reactions we have found showed significant variation depending on the CO_2_ concentration and confirmed effects on different metabolic pathways, such as glycerolipid synthesis, amino acid synthesis, glycolysis, carbohydrate catabolism, and carbon fixation, among others (Data S2). We assessed the ability of this model to predict growth rates by performing simulations under photoautotrophic, heterotrophic, and mixotrophic conditions. Fluxes of carbon dioxide, acetate, and light were adjusted according to the experimental evidence (Chang et al., 2011; Chaiboonchoe et al., 2016) to predict growth rates in the model (Table 2). Our results showed a significant coherence between conditions when compare the experimental and the *in silico* growth rates, with the lowest growth rate under heterotrophic conditions and the highest under the mixotrophic condition, which is in agreement with several previous reports (Moon et al., 2013; Imam et al., 2015). The growth rates predicted by FBA in terms of the CO_2_ and acetate input fluxes are shown in Fig. S1. The model predicts optimal values of acetate input fluxes close to 100 mmol/gDW h, without restriction of ATP production in the mitochondria or the chloroplasts. We have found that ATP production is an important restriction for growth that is dependent on environmental conditions. Response surfaces of growth rates predicted by FBA with respect to fluxes of acetate and CO_2_ and the reactions of ATP production in the mitochondria and chloroplasts are shown in Fig. 5. Simulation of the different growth conditions required blockade of ATP production in mitochondria under photoautotrophic conditions, blockade of ATP production in the chloroplast under heterotrophic conditions, and activity of both reactions under mixotrophic conditions. FBA suggested that at all tested conditions (i.e., heterotrophic, photoautotrophic, and mixotrophic conditions), the metabolic pathways related to photorespiration persist active, which fulfilled the model constraints.

**Table 2:** Growth rates predicted, and input fluxes of CO2 and acetate upon photoautotrophic, heterotrophic, and mixotrophic conditions.

**Components**	**Photoautotrophic**	**Heterotrophic**	**Mixotrophic**
CO_2_ Flux (mmol/gDW h)	−12	85,73	−12
Acetate Flux (mmol/gDW h)	0	−10	−10
µmax (1/h)	0,313	0,522	0,835

The magnitude of the fluxes for glycine transport from chloroplasts to mitochondria, serine transport from mitochondria to chloroplasts, and production of hydroxy pyruvate, glycerate, and 3-phosphoglycerate in the chloroplast showed positive values. These routes contribute to the maximization of biomass production because they are related to photorespiration processes (Kebeish et al., 2007). FBA revealed an increase in the flux for metabolic pathways happening in the chloroplast and mitochondrial transport systems in response to CO_2_ input variations as previously reported (Winck et al., 2016). Moreover, our results suggested that cells at high CO_2_ (photoautotrophic and mixotrophic conditions) have the ability to increase their biomass production.

## Discussion

The main goal of this study was to understand the biological response of *C. reinhardtii* to environmental changes in CO_2_ concentrations from a metabolic dynamics perspective. For this reason, we performed a sensitivity analysis to provide a reference for optimizing biomass production in response to variations in heterotrophic, photoautotrophic, and mixotrophic growth conditions by changing the carbon sources available in the extracellular medium. We also wanted to test the effects of changing the initial concentration of inorganic compounds, such as photons and water, in the extracellular medium to promote the photosynthesis process and biomass production. Some reports on the energy requirements for photoautotrophic growth of *C. reinhardtii* indicated that, at high light supply, the biomass yields decrease due to light saturation effects (Kliphuis et al., 2012). Light saturation effects also have been shown to be depended on DIC availability (Chang et al., 2011).The optimal value between low and high light supply still has to be elucidated, which represents an important future work.

**Figure 6:**
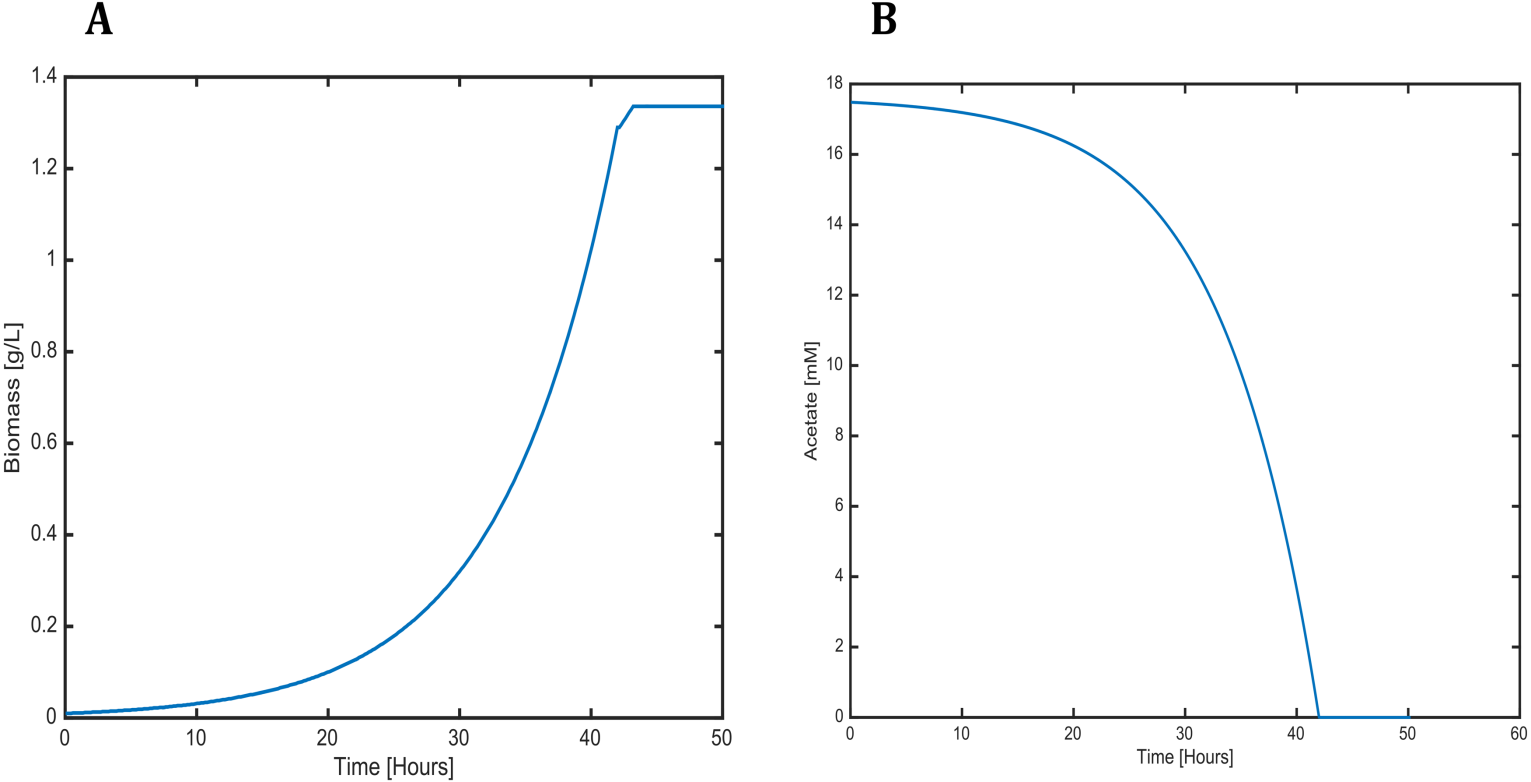
Profiles predicted by SOA of (A) biomass production and (B) acetate consumption over time.

**Figure 7:**
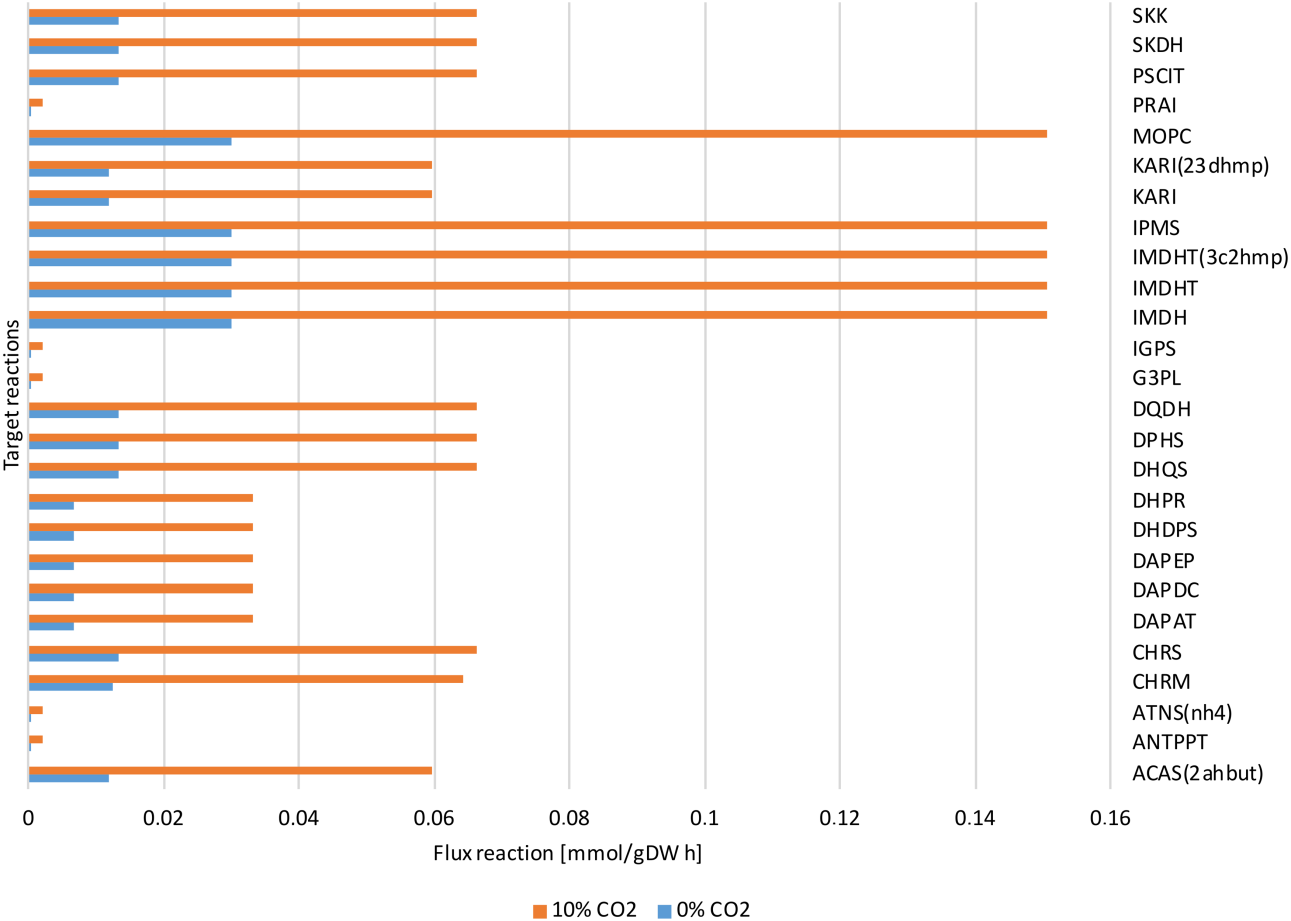
Reactions fluxes predicted associated to amino acids biosynthesis. Valine, leucine and isoleucine biosynthesis: (ACAS 2ahbut) acetolactate synthase, (IMDH) 3- isopropylmalate dehydrogenase, (IMDHT) 3-isopropylmalate dehydratase, (IPMS) 2- isopropylmalate synthase, (KARI) ketol-acid reductoisomerase, (MOPC) 4-Methyl-2- oxopentanoate conversion. Phenylalanine, tyrosine and tryptophan biosynthesis: (ANTPPT) pyrophosphate phosphoribosyl-transferase, (ATNS nh4) Anthranilate synthase, (CHRM) Chorismate mutase, (CHRS) Chorismate synthase, (DHQS) 3-dehydroquinate synthase, (DPHS) 3-deoxy-7-phosphoheptulonate synthase, (DQDH) 3-deydroquinate dehydratase, (G3PL) D-glyceraldehyde-3-phosphate lyase, (IGPS) indole-3-glycerol-phosphate synthase. Lysine biosynthesis: (DAPAT) diaminopimelate aminotransferase, (DAPDC) diaminopimelate decarboxylase, (DAPEP) diaminopimelate epimerase, (DHDPS) dihydrodipicolinate synthase, (DHPR) dihydrodipicolinate reductase.

The dynamic predictions using SOA agreed with the experimental data of biomass production where, at day three of cultivation, we obtained a biomass concentration of 1.3 g/L. The current reconstructed model used the same achieved values of the SOA, for less cultivation time, with the improved biomass production showing consumption of both carbon sources. Our model predicted mixotrophic growth accurately: acetate is used initially, but as acetate is consumed and its concentration is reduced, the CO_2_ consumption starts (Fig. 6). Our model was able to predict the behavior of variations in the synthesis of some amino acids (lysine, glycine, leucine, tyrosine, isoleucine, valine, and phenylalanine); some of these are biomass precursors and showed the most prominent alterations in response to high CO_2_ concentrations (Fig. 7). In the same way, our dynamic model predicts the fluxes of important transport reactions that involved relevant cofactors and substrates of the TCA cycle and carbon fixation mechanism. These were activated pathways mainly associated with the chloroplast and mitochondria and included hydroxypyruvate, 3-phosphoglycerate, ribulose 1,5-biphosphate, aspartate, and phosphoenolpyruvate production. All these processes may be related to photorespiration and showed positive values in our current reconstructed model. All these routes were shown to enhance biomass production under mixotrophic conditions. It is important to mention that CO_2_ will only increase the biomass if the cells are not limited for other nutrients like N or P.

### Metabolomic and transcriptomic data

We performed a canonical discriminant evaluation of the experimental data to compare with our dynamic modeling results. The canonical discriminant analysis allows evaluation of the presence of significant differences between groups of a set of measured variables in order to classify them. Figure S2 presents the canonical discriminant analysis grouping by growth condition alone. Figure S3 shows the canonical discriminant functions analysis obtained after grouping by both time and growth condition. A substantial discrimination was revealed by the opposite ranges of scores (Fig. S2), suggesting that growth condition is a function that discriminates in a proper way. In Fig. S3, the centroids show the CO_2_ groups in culture (0%-heterotrophic, 10%-mixotrophic) considering the metabolic abundance. Metabolites and abundance were shown to be significantly affected by changes in CO_2_ concentration level. Our dynamic modeling agreed with these results since it showed a significant increase of flux in metabolic routes including the TCA cycle, glycolysis/gluconeogenesis, and amino acid biosynthesis at high CO_2_ concentrations, as confirmed by the experimental abundance of metabolites involved in these pathways. Our data suggested that high CO_2_ concentration may activate mechanisms that are able to control carbon fixation.

The dynamic model predicted the profile only for the extracellular metabolites, so this required obtaining the fluxes of the reactions that mainly contributed to the experimentally observed metabolic abundance in order to make appropriate comparisons. The metabolomic data primarily showed the presence of amino acids and few sugars; therefore, to compare these data with the results predicted by our model, we primarily analyzed reactions involved in amino acid biosynthesis. Figure 7 shows the predicted reaction fluxes associated with amino acid biosynthesis, where a differential behavior was clearly observed between growth conditions (high values for reaction fluxes at high CO_2_ concentrations and low values for reaction fluxes at low CO_2_ concentrations). This is in agreement with the experimental results of metabolic abundance that showed opposing behavior between the two CO_2_ concentrations. This suggests that our proposed metabolic network model is highly sensitive and predictive of environmental changes in both steady-state and dynamic conditions.

**Figure 8:**
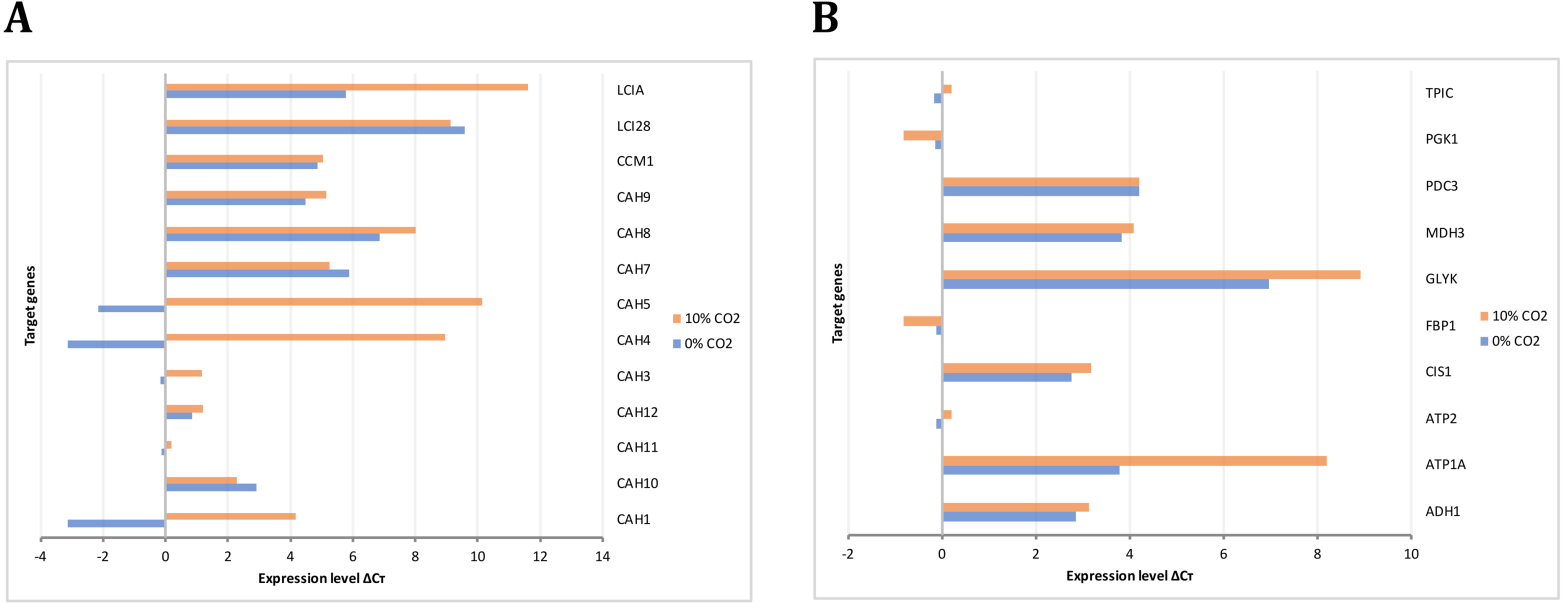
Experimental gene expression profiling. (A) Carbon concentrating mechanism: Carbonic anhydrases (CAH1, CAH3, CAH4, CAH5, CAH7, CAH8, CAH9, CAH10, CAH11, CAH12), Carbon concentrating mechanism 1 (CCM1), Aldolase reductases (LCIA, LCI28) (B) Glycolysis/Gluconeogenesis and TCA cycle: Alcohol dehydrogenase/acetaldehyde dehydrogenase (ADH1), ATP synthases (ATP1A, ATP2) citrate synthase (CIS1), fructose-1,6-bisphosphatase (FBP1), glycerate kinase (GLYK), NAD-dependent malate dehydrogenase (MDH3), pyruvate decarboxylase (PDC3), phosphoglycerate kinase (PGK1), triose phosphate isomerase (TPIC).

**Figure 9:**
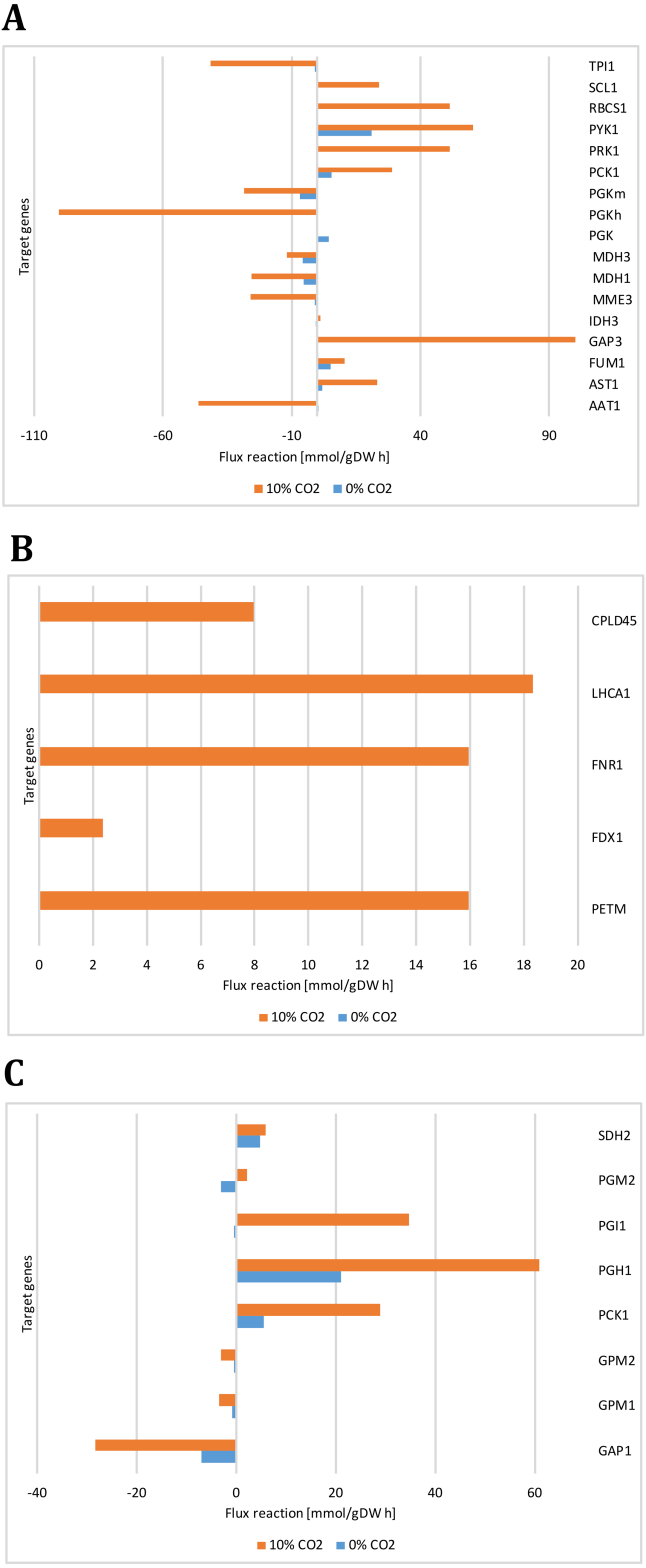
Reactions fluxes predicted for target genes associated with (A) carbon concentrating mechanism (B) photosynthesis, (C) glycolysis/gluconeogenesis and TCA cycle. Alanine aminotransferase (AAT1), aspartate aminotransferase (AST1), fumarate hydratase (FUM1), glyceraldehyde-3-phosphate (GAP3), isocitrate dehydrogenase (IDH3), malate dehydrogenases (MME3, MDH3), phosphoglycerate kinases (PGK, PGKh, PGKm), phosphoenolpyruvate carboxykinase (PCK1), phosphoribulokinase (PRK1), pyruvate kinase (PYK1), ribulose-bisphosphate carboxylase (RBCS1), succinyl-CoA ligase ADPforming (SCL1), triosephosphate isomerase (TPI1), cytochrome b6 (PETM), cyclic Electron Flow (FDX1), ferredoxin reductase (FNR1), photosystem I (LHCA1), photosystem II (CPLD45), glyceraldehyde 3-phosphate dehydrogenase (GAP1), phosphoglucomutases (GPM1, GPM2), glucose-6-phosphate isomerase (PGI1), phosphoglycerate mutase (PGM2), succinate dehydrogenase ubiquinone (SDH2).

The gene expression analysis performed by RT-PCR allowed the identification and relative quantification of genes involved in the carbon concentrating mechanism, TCA cycle, glycolysis, and gluconeogenesis. The expression level of those genes encoding carbonic anhydrases is shown in Fig. 8A. Here, we analyzed these genes since the previous evidence was indicating that they were of relevance in carbon uptake and biomass accumulation (Winck et al., 2016). Our results showed that gene transcripts for CAH1, CAH4, CAH5, and LCIA were more abundant at high CO_2_ concentrations, in agreement with previous reports of carbon uptake mechanisms in *C. reinhardtii* (Brueggeman et al., 2012)*.* Fig. 8B presents the expression levels of genes involved in glycolysis/gluconeogenesis and some of TCA cycle. Genes encoding for glycerate kinase (GLYK), ATP synthase alpha subunit (ATP1A), fructose 1,6-biphosphatase (FBP1) showed more abundant gene transcripts at high CO_2_ concentration. On the other hand, genes encoding for pyruvate decarboxylase (PDC3), NAD-dependent malate dehydrogenase (MDH3) did not show a differential behavior. These results are in agreement with our steady-state and dynamic simulations, which showed abundant gene transcripts in crucial genes involved mainly in carbon uptake mechanisms and the TCA cycle. Furthermore, they were related to the variation in differential growth conditions, which was confirmed in the fluxes of these reactions (including transport and exchange reactions) in our current reconstruction model (Fig. 9 and Data S3).

Our predicted results led us to consider new target genes that own the crucial plasticity needed to support growth adaptability. Figure 9 shows the reaction fluxes predicted for other target genes associated with the carbon concentrating mechanism, photosynthesis, glycolysis/gluconeogenesis, and the TCA cycle. It is important to note that we assumed that the reaction flux is directly related to the transcript abundance, given the enzyme activity, so differential behavior is clearly observed between growth conditions. Our predicted results suggest that transcripts for GAP3 (glyceraldehyde-3-phosphate dehydrogenase), RBCS1 (ribulose-bisphosphate carboxylase), PGK (phosphoglycerate kinase), PGI1 (glucose-6-phosphate isomerase), and PGH1 (enolase) must be more abundant at high CO_2_ concentrations. In the same way, our predicted results showed high and unique expression of genes related to photosynthesis under photoautotrophic and mixotrophic growth conditions. We have found that ATP production is an important restriction for growth, which depends on environmental conditions, in agreement with previous flux balance analysis in metabolic networks (Serrano-Bermúdez et al., 2017). This result suggests that our proposed metabolic network model could give complementary and high-value perspectives on these transcriptional changes.

## Conclusions

In this work, we have significantly improved the previous models, obtaining a new robust genome-scale metabolic network model for the microalga *Chlamydomonas reinhardtii.* Our current model is appropriately curated and lacks any thermodynamically infeasible cycles, and it is highly sensitive to environmental changes in both steady-state and dynamic conditions, with kinetic parameters associated with substrate consumption at different growth conditions (0% CO_2_-heterotrophic and high CO_2_-mixotrophic). The experimental results agreed with steady-state and dynamic simulations, showing abundant gene transcripts in crucial genes involved in carbon uptake mechanisms and the TCA cycle. Our proposed metabolic network model can, therefore, provide complementary and high-value perspectives on transcriptional changes, metabolic abundance, and biomass production under different environmental conditions. Finally, up to now, no reports exist regarding dynamic flux balance analysis in *C. reinhardtii*. Consequently, our results and predictions can improve the understanding of the biological response of *C. reinhardtii,* as a dynamic entity, to environmental changes. This is of great interest given the biotechnological potential of this organism for CO_2_ fixation, biomass accumulation, and bioenergy production.

Model predictions with FBA allows the description of different metabolic states regarding the variations in the growth conditions. In addition, the dynamic approaches have revealed a robust model with a high predictive capacity, which is a useful tool to further analyze *a priori* effects of several perturbations on the culture media, limiting factors, metabolic pathways, potential mutations, biomass production, lipids accumulation, and so on. Thus, our model represents a useful contribution to the field since it will allow to propose, analyze and evaluate different conditions affecting biological systems from a dynamic point of view. This is of relevance as biological systems are highly dynamic entities affected by a wide variety of changes in the environment, as a part of an adaptation process.

##  Supplemental Information

10.7717/peerj.5528/supp-1 Figure S1 Response surface of growth rates predicted by FBA in function of CO2 and acetate fluxes [mmol/gDW h]

10.7717/peerj.5528/supp-2 Figure S2 Canonical discriminant analysis of metabolomic data (A) heterotrophic growth condition (0% CO2) (B) mixotrophic growth condition (10% CO2)

10.7717/peerj.5528/supp-3 Figure S3 Canonical discriminant functions analysis of metabolomic data (A) grouping by time (B) grouping by growth condition and time

10.7717/peerj.5528/supp-4 Data S1 New comprehensive genome-scale network reconstruction for *C. reinhardtii*, which consisted of 3726 reactions and 2436 metabolites

10.7717/peerj.5528/supp-5 Data S2 New comprehensive genome-scale network reconstruction for *C. reinhardtii*

10.7717/peerj.5528/supp-6 Data S3 Effects on different metabolic pathways, such as glycerolipid synthesis, amino acid synthesis, glycolysis, carbohydrate catabolism, and carbon fixation, among others

## Additional Information and Declarations

### Data Availability

The following information was supplied regarding data availability: The raw data are included in the Supplemental Files.
