# Supplementary figures and images for: Development of a Chlamydomonas reinhardtii metabolic network dynamic model to describe distinct phenotypes occurring at different CO2 levels
Source: PeerJ. 2018 Sep 3;6:e5528. doi: 10.7717/peerj.5528 (PMC6126472; doi:10.7717/peerj.5528)

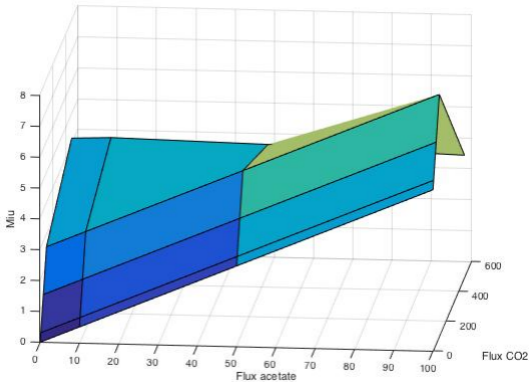

Supplement: Figure S1 [file peerj-06-5528-s001.pdf]

A

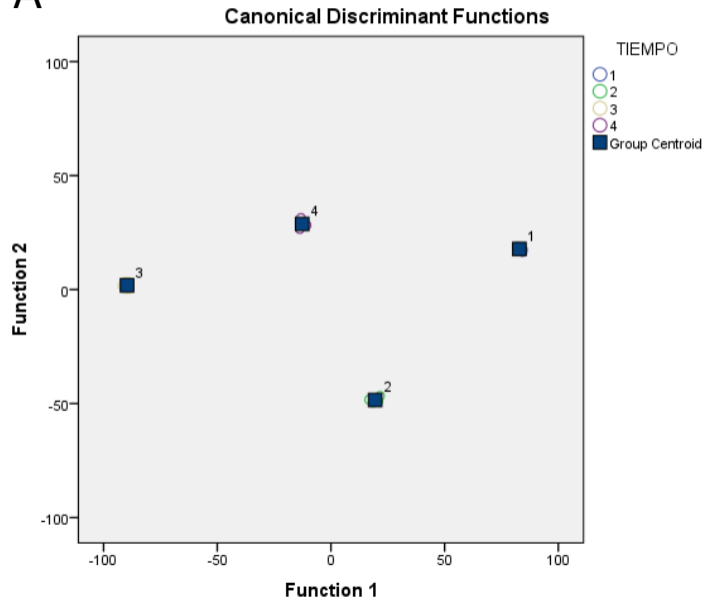

B

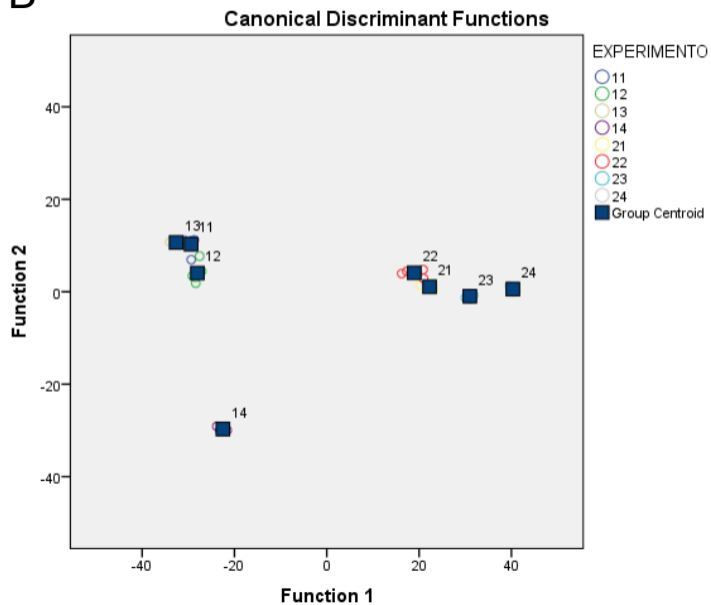

Supplement: Figure S3 [file peerj-06-5528-s003.pdf]
