# Supplementary material for: Development of a Chlamydomonas reinhardtii metabolic network dynamic model to describe distinct phenotypes occurring at different CO2 levels
Source: PeerJ. 2018 Sep 3;6:e5528. doi: 10.7717/peerj.5528 (PMC6126472; doi:10.7717/peerj.5528)
Supplement: Figure S2 [file peerj-06-5528-s002.pdf]

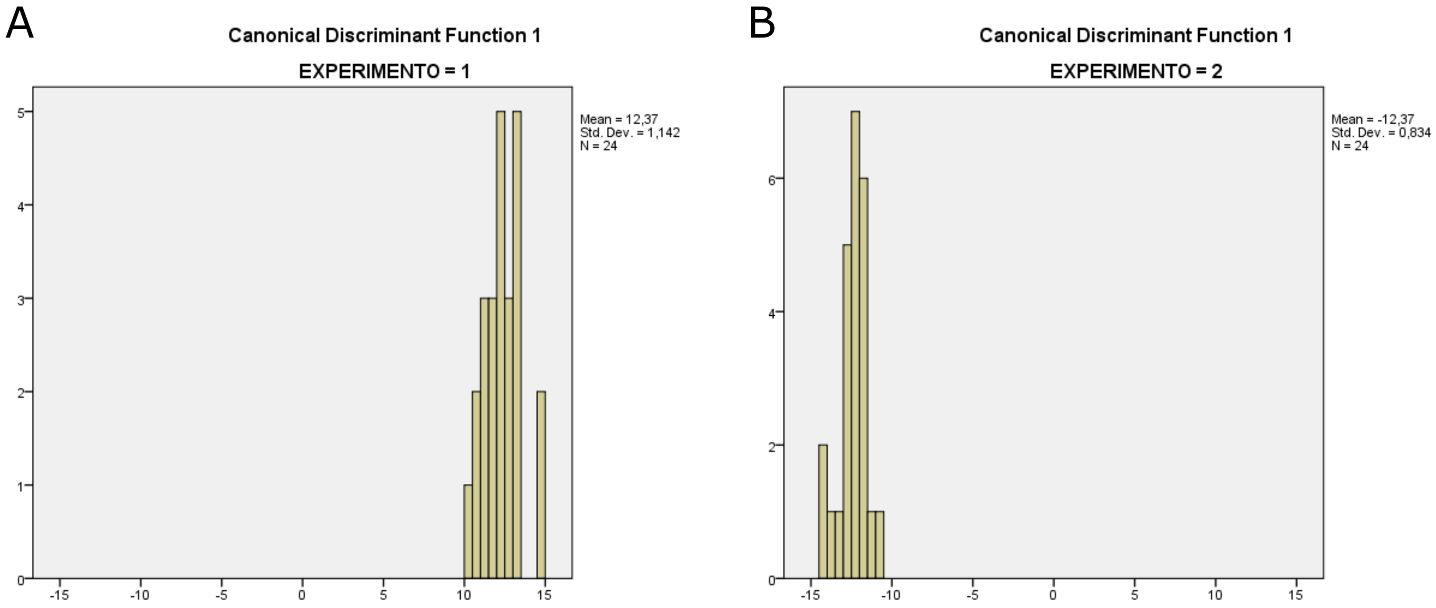
A
Canonical Discriminant Function 1
EXPERIMENTO = 1
Mean = 12,37
Std. Dev. = 1,142
N = 24
B
Canonical Discriminant Function 1
EXPERIMENTO = 2
Mean = -12,37
Std. Dev. = 0,834
N = 24
